# Implementation of a Recovery College Embedded in a Swedish Psychiatry Organization: Qualitative Case Study

**DOI:** 10.2196/55882

**Published:** 2024-09-12

**Authors:** Lina Al-Adili, Moa Malmqvist, Maria Reinius, Inka Helispää Rodriguez, Terese Stenfors, Mats Brommels

**Affiliations:** 1 Medical Management Centre Department of Learning, Informatics, Management and Ethics Karolinska Institute Stockholm Sweden

**Keywords:** mental health, educational intervention, recovery college, implementation research approach, qualitative research, coproduction

## Abstract

**Background:**

Recovery colleges are service user–led educational interventions aiming at empowering people with mental health issues and promoting recovery through peer learning. Despite the increasing interest in recovery colleges in recent years and the demonstrated beneficial effects for users, there is limited research addressing aspects that influence their implementation. This knowledge is necessary for the successful integration of such interventions in various contexts.

**Objective:**

This study aims to explore factors that influence the implementation of a recovery college embedded within a Swedish psychiatry organization.

**Methods:**

A qualitative case study of a recovery college based on semistructured interviews with 8 course participants, 4 course leaders, and 4 clinical staff was conducted. The transcripts were scrutinized with conventional content analysis, and the interpretation of results was guided by the Consolidated Framework for Implementation Research.

**Results:**

The findings highlight key areas that either hinder or promote the successful implementation of the recovery college. These areas included recruitment, resources, staff attitudes, and ways of organizing courses. Each area has elements that appear both as facilitators and barriers, demonstrating the duality of conditions.

**Conclusions:**

Allocating dedicated resources, engaging individuals with service user experience as organizers who are willing to share their personal experience, having an open-door policy, creating an open space for participants to share their experiences, and offering practical advice and written material are useful to create favorable conditions for a recovery college to reach its goals of empowering psychiatry service users.

## Introduction

### Background

Many mental health service users have engaged in self-care with the aim of taking control over their lives despite diseases as well as turning to peer support. This started in the United States as a mental health consumer movement in the 1970s. In an *emerging issues* paper, Davidson [1] discussed how this movement has been supported by changes in US legislation from the 1990s onward. He referred to longitudinal studies of patients with schizophrenia performed in the 1970s and 1980s that changed the previously pessimistic view on psychiatric disorders. This was well in line with the personal experience of people that they were, despite a psychiatric diagnosis, able to lead meaningful and productive lives. He also claimed that those studies showed that the capacity of patients to recover fully or learn to manage their condition, in many instances developed outside formal treatment settings.

Perkins et al [2] differentiated this patient-driven self-management activity from professional psychiatric care by referring to those approaches as *educational* versus *therapeutic approaches*. Instead of focusing on problems and dysfunctions and labeling all activities as therapies, the recovery movement supports people to identify and develop their talents and skills, explore their possibilities, and focus on achieving ambitions and goals. It has, consequently, also been defined as an *assets-based* approach, aiming at developing the *recovery capital* of patients, defined as “the array of social, psychological and cultural networks beyond professional inputs” [3].

“Recovery colleges” are such educational activities that have proliferated in the United Kingdom. A network titled *Implementing Recovery through Organisational Change* coordinates about 40 recovery colleges that engage over 500 peer workers, promoting learning and self-management as core practices among patients with mental health conditions [4]. Recovery colleges are typically led by persons with lived experience as service users and they focus on sharing experience, support for coping, and skills training.

The interest in recovery colleges has increased over the years, more colleges have been established, and the number of reports on their outcomes keep growing. A recent systematic review concluded that “Recovery college attendance was associated with high satisfaction among participants, attainment of recovery goals, changes in service providers’ practice, and reductions in service use and cost” [5]. Attending a recovery college was described by participants as being useful in supporting recovery, leading to a decrease in service use [6]. Another study reported that well-being and personal resources were strengthened, and user satisfaction increased as the service provided was perceived as accepting and enabling. In addition, participants felt a greater sense of hope, confidence, and higher aspirations [7]. In focus group interviews, recovery college participants expressed that they had experienced a positive impact on their lives and had seen benefits brought by the college to the organization [8].

A systematic literature review analyzed outcomes of recovery college activities on mental health staff, mental health services, and the society at large [9]. Mental health clinical staff who participated in recovery colleges valued collaboration with service users and, as a result, gained a different perception of those service users and felt more passion and higher job motivation. Within mental health organizations, recovery college activities provided staff with a learning environment to practice coproduction with users. Recovery colleges involve agencies in the community and their staff in collaboration with service users, which has a positive effect on staff attitudes and public opinion [9].

Some impact studies have included process evaluations with information on program content and resources used. Those tend to focus on improvement opportunities, such as standardizing course processes and planning for longer courses [10]. Hall et al [11] represented 1 group of a few researchers addressing the implementation of a recovery college. They found “delays in the development of some key policies and procedures, including the enrollment and attendance information, standardization of evaluation measures and course standardization” [11]. The reasons for these delays were lack of resources, funding, and staffing; staff turnover; and less defined staff roles. Some staff felt uncertain about coproducing with persons with lived experience and the quality of external expert input. Slade et al [12] found similar attitudinal problems among staff, characterizing those as “abuses of recovery colleges.” Staff might feel that recovery colleges are a fad, that those would not benefit their patients, and that psychiatry services would be sufficient to address their problems.

In summary, these studies on the outcomes of recovery college activities show high satisfaction among participants, experiencing a greater sense of hope, confidence, strengthened personal resources, and a positive impact on their lives in general. Finally, participants had reduced their use of formal services. Mental health professionals with experience in recovery colleges valued collaboration with service users and reported, as a result, feeling more passion and higher job motivation. The collaboration between recovery colleges and agencies in the community had a positive effect on the staff of those agencies and public opinion. However, some challenges were also reported. Lack of resources, funding, and staff attitudes would delay the launch of a recovery college. Some staff members felt that the activity would not benefit their patients beyond that of formal psychiatry services.

When setting up a recovery college, prospects for success would be enhanced by a clear conceptualization of the college, an integration between the college and the host organization, and attention paid to the power imbalance between providers and patients [13]. These observations refer mostly to the design of the educational activity, whereas information on the way in which plans have been carried out and adjusted to fit local conditions and contexts is lacking. Such approach is referred to as *implementation*, which preferably should be studied with an *implementation research approach* [14]. Hence, implementation includes not only the introduction of an intervention but also the continuous adaptation and optimization of it within the organizational context.

### This Study

Given the scant literature and the importance of understanding the context, we set out to specifically study the *implementation* of a recovery college that is embedded in a psychiatry organization. Elsewhere, recovery colleges are typically freestanding centers. We took advantage of the fact that we had access to 1 recovery college at a psychiatry clinic, called *Patient School*, in Region Stockholm, Sweden. We have recently analyzed the value of this Patient School, as described elsewhere [15]. Hence, the aim of this study was to explore factors that influence the implementation of the Patient School within this psychiatry organization.

## Methods

### Study Design

This is a qualitative inductive study based on semistructured interviews conducted using a coproduced approach [16,17]. The research team included persons with formal experience of research (health care professionals and other academically trained individuals), those with lived experience of being a patient in a mental health care facility, and those presently working in the psychiatry organization. The team of authors cocreated all different aspects of the research process, including reflexive discussions on how team members’ different perspectives have affected the research process. The COREQ (Consolidated Criteria for Reporting Qualitative Research) guidelines have been followed to support the transparency and quality of this research [18]. To strengthen the focus on the implementation process, the analysis and the interpretation of the data were guided by the updated Consolidated Framework for Implementation Research framework, as proposed by Damschroder et al [19].

### Context

The psychiatry organization provides both inpatient and outpatient services to the Region Stockholm population and is part of its public health care. It has consistently led efforts in fostering user participation and organizing user-centric initiatives within the mental health sector of this region. Since 2007, the psychiatry organization has appointed dedicated *user-involvement coordinators* on a full-time basis. By 2016, the organization expanded its approach by incorporating peer-support workers, known as *staff with user experience*, who serve as mentors for patients in psychiatry units. User-involvement coordinators conduct regular surveys among users to gather insights and relay this information to the psychiatry organization’s management. In addition, a user-involvement coordinator holds a position in the organization’s Patient Safety Group and presides over the User Council, which includes members from patient organizations and the management team. The founders of the Patient School were working within the organization as user-involvement coordinators or staff with user experience. The Patient School was established in 2018 by the user-involvement coordinators and offered initially to outpatient users. The clinical manager, who the lead user-involvement coordinator reported to, endorsed the plan and anchored it with the full senior management team of the organization. The Patient School gatherings take place in psychiatry care facilities with the support of the management and with professional staff contributing.

As guiding principles for the Patient School, they agreed upon (1) promoting recovery; (2) placing the activity in facilities within the psychiatry organization with the support of its leadership; (3) choosing employed user-involvement coordinators and staff with user experience as coordinators; and (4) while encouraging sharing of personal experience, avoiding suggesting those as generalizable recommendations.

Before launching the first Patient School program, the course leaders had visited recovery colleges in England, acquiring inspiration from that experience. They then formed a working group to ensure they all had the same vision for the program. All leaders were present at every meeting during the first round of Patient School so that they would all teach the course the same way. After that, the work was divided, and leaders were assigned sessions with specific themes so that not all leaders had to be present every time.

As previously described by Reinius et al [15], the Patient School was founded in 2018 for both inpatient and outpatient units. However, information about the Patient School was originally circulated at outpatient departments (ambulatory mental health centers). All participants so far have been recruited this way.

In total, 12 courses were offered, with close to 70 course participants. The Patient School consists of a series of five workshops offered over 5 weeks covering the following themes: (1) psychiatry: how does it work? (2) recovery: what is helpful? (3) other resources in society, (4) relations and disclosure, and (5) personal tools. The course leaders invited, to each workshop, health care personnel from the psychiatry organization or researchers to act as coleaders and substance matter experts.

The study is part of the *Patients in the driver’s seat* partnership research program, situated at Karolinska Institute exploring patient-driven innovations to promote self-care and cocare [20].

The choice of themes to include in the course curriculum was based on views expressed by psychiatry service users in *Patient forums,* organized by the user-involvement coordinators planning the Patient School. Some of those were related to *patient competence*, that is, knowledge about the health care system and laws and regulations needed to be able to *navigate the system*. Patient School participants (service users) were asked for feedback, both orally and in surveys, and the content was adjusted accordingly. Participants in previous courses were engaged to be mentors to new participants and participated alongside them. These mentors shared their observations and gave useful feedback.

### Participant Recruitment

The data used for this study were gathered as part of a larger research project as described in the study by Reinius et al [15]. In total, 45 participants in the Patient School who had provided contact information during or after completing the school were invited by MR to participate. In total, 7 clinical staff who acted as experts as well as 6 course leaders (user-involvement coordinators and staff with user experience) were also sent invitations. Apart from one who is a coauthor with user experience (IHR), no previous relationships with IHR were established before the commencement of the study. MR was introduced as a researcher interested in exploring participants’ views about the Patient School. The timeline of respondent recruitment is presented in Multimedia Appendix 1.

### Ethical Considerations

Ethics approval was granted by the Regional Ethical Review Board of Stockholm (Dnr 2019-03849 with amendment Dnr 2020-04604). All procedures followed were in accordance with the ethical standards of the responsible committee for human experimentation (institutional and national) and with the Helsinki Declaration of 1975, as revised in 2000. Informed consent was obtained from all patients for being included in the study. The data are anonymized, and no compensations were provided to participants in this study.

### Data Collection

A researcher trained in qualitative methodology and interview technique was responsible for developing a semistructured interview guide, and it was discussed, revised, and received approval from the entire team. The interviews were conducted over the telephone by the same researcher MR from her office. The interviews were recorded and transcribed verbatim. The respondents had received written information in advance and were able to ask questions before the interview started. An interview guide was designed in discussions within the research team, including members who had been involved as course organizers. Their experience was important in identifying different items of the implementation process that could be used in follow-up questions. However, the interviews started with open-ended questions, such as “according to you, what is needed for the Patient School to be carried out? and probes such as can you tell me more about that?” The data collection stopped when no more aspects connected to the study aims were identified, that is, when data saturation was reached.

### Data Analysis

The transcripts were subjected to conventional content analysis using an inductive approach [21]. For this manuscript, interview data were analyzed with particular focus on aspects of implementing the Patient School. First, MR read through all transcripts several times to reach immersion and formulated meaning units to cover all sections of the text that responded to the aim and defined 2 main themes (ie, barriers and facilitators). Barriers refer to obstacles and difficulties when organizing courses, and facilitators refer to conditions that make implementation easier or promote perceived successes. MB read 5 transcripts to verify the preliminary categorization.

The selected meaning units were checked against the original transcript, labeled, grouped, and posted on a Miro dashboard by MM. MB, MMC, TS, and IHR participated in 4 analysis workshops that started with all participants reading the meaning units in silence and making notes on their first impressions, thoughts, and initial analysis. The preliminary labeling and categorization were discussed in the full team, and agreement was reached on defining subcategories. All authors reviewed initial findings and suggested revisions until a consensus was reached. MMC then returned to the full data related to the selected meaning units to select representative citations. To validate those, LA read all the transcripts and confirmed the preliminary analysis. In this way, data analysis was performed by all team members participating while also protecting the integrity of the interviewees. As it was felt that member checking would have run the equal risk of individual interviewees being identified, the procedure was not performed.

LA was responsible for manuscript writing and composition. She drafted and revised the manuscript based on critical input from the other authors. Of crucial importance were user-involvement coordinator members’ comments, which guided the contextual interpretation. All authors approved the final manuscript.

## Results

### Overview

In total, 16 individual interviews were conducted from March to May 2021 (lasting between 25 min and 75 min) with 8 (50%) course participants, 4 (25%) course leaders, and 4 (25%) clinical staff who had participated in the Patient School as invited experts.

The findings highlight key areas that either hinder (barriers) or promote (facilitate) the successful implementation of the Patient School within the psychiatry organization. These areas encompassed *recruitment*, *resources*, *staff attitudes*, and *ways of organizing courses.* The findings are structured around these distinctive subthemes. Each subtheme appears both as a facilitator and a barrier, demonstrating opposite conditions. Our comprehensive summary of the findings is described in Textbox 1.

**Summary of barriers and facilitators for the implementation of the Patient School based on interviews with course leaders, participants, and staff and course documents.**

**Barriers**

- Recruitment
  Lack of contact with fellow service users
  Lack of knowledge and understanding of the Patient School and its benefits among clinical staff
- Resources
  Patient School not included in the reimbursement system
  Focus on service production and less time for staff to support Patient School
  Lack of a dedicated venue
- Negative attitude among staff
  Negative stance toward staff with user experience and patient involvement
  Change resistance—fear of heavier workload
*Wrong to teach a person to be a patient*
- Ways of organizing course
  Course leaders spending too much time describing their own experience left little space for participants
  Some participants dominated too much
  Some experts not appreciated by participants

**Facilitators**

- Recruitment
  Everybody can join the Patient School
  Active information to patients from staff
- Resources
  User-involvement coordinators and staff with user experience as course leaders
- Positive attitude among staff
  Patient satisfaction and perceived value of Patient School increases staff motivation to support Patient School
- Ways of organizing course
  Course leaders sharing their own experience encouraged participants
  Moderator giving everybody space
  Participant feedback paid attention to
  *Open door policy* (everybody is welcome)
  Appreciated course material

### Recruitment

Recruitment barriers for the Patient School were primarily attributed to limited contact between patients and staff with user experience as well as user-involvement coordinators and inadequate information dissemination by staff. The staff were described to have an essential role in recruiting patients and conveying the value of the Patient School. Participants acknowledged that not all patients had the opportunity to meet with staff with user experience and user-involvement coordinators directly, highlighting the importance of regular staff interactions with patients to disseminate information about Patient School and assist in recruitment efforts:

[In order for the patient school to be implemented, it is necessary] that [staff] want to participate, of course. Participate both with us and to help get information out so that people will be interested in it. So a collaboration is required.

Interviewee #10

Ensuring that information about Patient School was available in wards and outpatient departments was described to be essential for successful recruitment. Although written materials were accessible in the clinics, participants viewed verbal reminders by staff as a necessary complement. However, the lack of active information about Patient School to patients from staff was described as a barrier by several participants. One staff interviewee explained that, although reminding patients about Patient School would be helpful, it was easily forgotten about.

Some participants highlighted a lack of knowledge and understanding about Patient School among other staff. Interviewed staff described uncertainty about its structure and a lack of adequate information about how to provide patients with information about Patient School. Consequently, this led to feelings of insecurity when discussing the Patient School with patients.

The lack of an information channel about the Patient School was believed to contribute to a low understanding of Patient School among staff. Course leaders believed that it was difficult to spread information about Patient School to staff and that it would have been valuable if information of Patient School benefits would have been shared with them. They expressed concern that patients who did not have the opportunity to meet with a user-involvement coordinator or staff with user experience might miss out on being informed about Patient School:

What can be an obstacle, then, is...that they, patients, have not met us, and are not informed by staff, i.e. their contacts at outpatient care, that the Patient School exists.

Interviewee #3

Recruitment was facilitated by adopting an inclusive approach, wherein all outpatients at the clinic who were willing and capable of participating in structured group events were welcomed to participate. It was also seen as a future enabling factor to further spread the Patient School across all clinics in the region. That was desired by both staff and course leaders and could help both increase the size of groups that were felt to be too small and minimize frequency of waiting lists, which sometimes occurred. It was also believed that if patients from other clinics were recruited, it would help spread the word about Patient School. However, some interviewed staff raised concerns about mixing participants from different stages of recovery in the same sessions. They believed that there was a risk that people who had progressed on their path to recovery might have a flashback. This was confirmed by 1 staff member:

Those who leads it [the Patient School] should have knowledge about whether there’s a participant there who if something comes up that makes them feel bad, or triggers a flashback...that they can handle it. I think that whoever it was that was leading it, was very receptive to how people were feeling and how they reacted to what was said. It’s important to have the right person leading it.

Interviewee #12

Some participants made suggestions for the future improvement of the Patient School and expressed appreciation for the attentiveness of the course leaders to their feedback. For instance, a proposal was made to link participants’ care plans with the course program, which could create added value. Another proposal was to involve former participants to visit the Patient School, share their experiences, and aid course leaders. Those alumni would shadow a course leader for some time to learn the dynamics of the Patient School and afterward contribute as assistants to a course leader.

### Resources

The success of Patient School was described as relying on essential resources, including the availability of user-involvement coordinators and staff with user experience, time, suitable venues, and funding. The integration of Patient School in the regional health care reimbursement system was seen as the most important promoting factor, and if it was not, Patient School would not be able to evolve, let alone survive. The absence of Patient School from the reimbursement system was thus highlighted as a significant barrier to its implementation:

But I think the priority would probably be to try to approach the clients or those who manage that part, and see if there is any order, some type of compensation we can get as a business, to hold the Patient School. Because I think it’s more essential for us to survive.

Interviewee #7

Participating course leaders described that with earmarked funding, more course leaders could be hired, which would increase the number of sessions, lecturers with care provision commitments could be recruited, and a spread of Patient School across clinics would be possible. Another improvement would be to include Patient School education as a service to be reimbursed, in parallel with clinical services. The lack of these preconditions contributed to an undersupply and a long waiting list for participants to join Patient School at the clinic.

Participating course leaders emphasized that, at present, Patient School is held in the clinic’s facilities and the venue must be booked in competition with other activities. Course leaders stressed the need for improved access to clinic facilities, of which some could be specifically dedicated to Patient School. When requesting the venue, course leaders were sometimes met with resistance, which was seen as a direct effect of Patient School not being a part of the reimbursement system. Patient School competes with other initiatives that generate income for the clinic, which often were given first access.

Course leaders explained that they needed more time allocated to Patient School and to planning Patient School workshops. Some described that a dedicated budget for hiring expert lecturers would ease the burden on course leaders. Other course leaders stated that almost all clinics have used user-involvement coordinators and highlighted that to expand Patient School to additional sites would require either allocation of more staff or more active collaboration between user-involvement coordinators.

### Staff Attitudes

Several barriers connected to staff and managers’ attitudes were highlighted by course leaders. Some described a noticeable reluctance among staff toward including staff with user experience in health care in general. As the Patient School was initiated by user-involvement coordinators and staff with user experience, this affected staff attitude toward Patient School. A drastic example of the consequence of a negative attitude was told by course leaders. On some occasions, staff falsely claimed to have reserved the facility where Patient School was to be held. This behavior was perceived by some course leaders as an indirect expression of staff’s doubts about the value of the Patient School. Course leaders felt that some managers also were critical of the Patient School and misunderstood its purpose:

Then there have been some attitudes...obstacles too. There have been certain...Some managers, who have thought that no, should you really teach people to be patients?

Interviewee #7

A viewpoint expressed by some course leaders was that managers appeared to prioritize financial considerations over quality aspects. They suggested that managers perceived Patient School as less significant, as it does not generate income for the provider.

According to course leaders, there existed a degree of reluctance among staff toward Patient School among some staff. They had experienced that staff had actively singled out aspects of Patient School to criticize. This attitude was felt to mirror the fear of an increased workload triggering change resistance. One staff interviewee stated that during Patient School sessions, patients were encouraged to actively engage in care planning and participate in their care, such as by reading their medical records:

There are people who believe it’s, unnecessary, to remind that one can read one’s medical record, I heard from a colleague once, since the patient had expressed concerns (about a note and its content). I believe it’s evident that patients should be able to read their medical record, and at the same time, also to use it as a tool, as I do. However, not everyone likes it...So, of course, it’s true that some find it worrying that...patients, are well-informed and also that they have demands.

Interviewee #10

In contrast, facilitators included the perceived value of the Patient School, which not only influenced the general staff attitude toward Patient School but was also said to impact their willingness to recruit patients to participate. Patient satisfaction with the Patient School was described as a motivating factor leading to the dissemination of information about the program. For example, 1 staff interviewee took the initiative to frequently remind colleagues to inform patients about Patient School. In addition, 1 course leader suggested that staff on some occasions should accompany their patients to Patient School workshops, allowing them to gain firsthand experience of the Patient School and realize its value.

### Ways of Organizing the Course

The role of course leaders and the collaboration between them and participants were widely acknowledged as a cornerstone of a successful Patient School. Among the challenges encountered was the issue of equal participation during discussions. Some participants recognized their tendency to dominate discussions, hence limiting contributions from more quiet peers. The role of course leaders was thus emphasized as vital to directing the discussion, introducing clear topics, and helping participants to maintain focus. One staff interviewee highlighted the importance of the course leaders’ competence in directing the conversation:

I believe they were very competent at leading...you need the right person to lead it, someone with knowledge who is responsive and can evaluate how the information is being received by participants...And could interfere if a participant started to talk too much...and quickly redirect the conversation.

Interviewee #10

Participants expressed their appreciation of the skills of course leaders as moderators and mentioned that they had high trust in them. Course leaders highlighted that they made sure that everyone had a chance to speak and that all topics were covered. By sharing their own experiences, course leaders encouraged patients to speak up. Those features were seen as facilitating the successful implementation of the Patient School. Conversely, the role and behavior of course leaders were sometimes described as a barrier. Initially, course leaders at times focused too much on sharing their own experiences. This trap was avoided by creating clear agendas for sessions. Furthermore, course leaders described that to enhance coherence and promote improved group dynamics the following policy was implemented: if a participant missed the 2 first meetings, they had to quit the course.

As employees of the psychiatry organization, course leaders knew what psychiatry has to offer. Having user experience, they also succeeded in presenting a balanced view of life. In addition, by countering negative stories with positive examples, they wished to provide a nuanced perspective on the life situation of a user, contributing to the perceived value of Patient School:

Course leaders try to balance each other with examples we take from our own lives. That if someone has a very negative experience of a single event...maybe someone else has a more positive picture. And then we sort of try to balance that with the fact that it can look different.

Interviewee #7

Participants shared various additional observations of a positive experience related to the Patient School. Participants expressed their satisfaction with the course material and believed that the 5 meetings, which had different foci fit well together and progressed in a logical order. They also valued the fact that course leaders were in the position to contact clinical staff and facilitate medical interventions when needed. The practice of course leaders working in pairs was also appreciated, as it enables the leader to have a private encounter with a participant when needed without disrupting discussions within the rest of the group. Furthermore, a guest lecturer providing expert insights was something described as beneficial. In contrast, on 1 specific occasion, a guest lecturer was critical of psychiatric care, which was considered less constructive.

### The Lens of an Implementation Research Framework

#### Overview

To further highlight the primary focus of the study, the implementation of the Patient School program, the Patient School was analyzed using the additional information provided in the context in relation to the five dimensions of the Consolidated Framework for Implementation Research [19]: (1) intervention characteristics as defined by the *content* of the Patient School, (2) its outer setting, (3) inner setting, (4) individuals, and the (5) implementation process.

#### Patient School Content

The aim of the Patient School was to promote recovery and to reach out to service users by placing itself in facilities within the psychiatry organization and to charge user-involvement coordinators and staff with user experience to organize and lead the school workshops.

Each school course consisted of five workshops offered over 5 weeks, titled (1) psychiatry: how does it work? (2) recovery: what is helpful? (3) other resources in society, (4) relations and disclosure, and (5) personal tools. Health care staff from the psychiatry organization and researchers were invited as either coleaders or subject matter experts.

#### Outer Context

The outer setting of the Patient School was the Region Stockholm, Sweden, a comprehensive psychiatry organization, covering in-hospital care as well as outpatient services. The commitment of the organization to use patient-centered practices and ensure user influence and involvement was shown by the employment of persons with user experience as part of the permanent staff.

#### Inner Setting

The inner setting was the outpatient departments offering facilities for inpatients and outpatients to join the Patient School, organized by the salaried staff with user experience. The school was backed by supporting clinical staff, informing them about the Patient School, and participating in the active recruitment of participants. A barrier was the lack of earmarked funding and dedicated venues.

#### Individuals

The individuals involved were high-level managers having instituted the functions of user-involvement coordinators and staff with user experience and supporting their various initiatives. The school organizers benefited from their own user experience as well as being salaried staff of the organization. Clinical staff that had positive attitudes to user involvement participated in recruiting participants as well as contributed with information and expert advice. Finally, service users were active participants, sharing their experiences, and supporting the continuous improvement of school activities by giving regular feedback.

#### Implementation Process

The implementation process was characterized by the school content, covering practical information on services and support available, as well as skills training, and the creation of a safe environment for sharing experience by the example of the course leaders. Success factors facilitating the implementation process were an *open door policy* psychiatry staff actively informing service users of the Patient School, the lived experience of the course leaders, positive attitudes among some professional staff, and course leaders’ attention to participant feedback. Barriers to successful implementation were a lack of dedicated resources, negative attitudes among some staff who had doubts about the benefits of the Patient School, and instances where course leaders or participants dwelled too much on sharing personal experiences, thus impeding an open discussion and reflection process.

## Discussion

### Principal Findings

In our study, focusing on the implementation of a recovery college–like Patient School organized by persons with user experience within a psychiatry organization, we identified activities and attitudes that had both positive and negative impacts, that is, that could be both hindering and promoting factors. In terms of *recruitment,* the lack of both knowledge about the Patient School among staff and contacts with user-involvement coordinators and staff with user experience were barriers, whereas staff actively informing potential participants, the information provided during other user-activating courses, and the open-door policy created opportunities to reach out to potential participants more broadly. As to *resources*, educational activities such as the Patient School were not included in the reimbursement scheme for the psychiatry organization and were consequently felt to compete with service provision generating income, thus reducing the possibility for staff to contribute and salaried staff with user experience to take on organizer duties. In contrast, dedicated funds for the Patient School would remove those barriers and make it possible to pay honorariums to external experts. A dedicated venue would also be helpful to course organizers. Negative *attitudes among staff* were demonstrated as a negative attitude toward employees with user experience and suspicions about the value of the Patient School, change resistance, and negative views on patient involvement and empowerment in general. Staff who saw evidence of the value of the Patient School had a positive attitude and recommended patients to join the school. The *ways of organizing* the school had negative as well as positive consequences. When course leaders spent too much time on their own experiences and let a single participant dominate the discussions, other participants felt uneasy. In contrast, those course leaders who shared their own experiences encouraged participants to express their own concerns. Course leaders who gave everybody space and paid attention to participant feedback were appreciated. However, some expert contributions being out of touch with Patient School principles were seen as disturbing, whereas the course material was assessed as proper and useful. In summary, course leaders, participants, and staff identified the following facilitators of successful implementation: active recruitment of participants at wards and outpatient departments, information freely available in the same locations, a dedicated budget and venue for course activities, active moderation of discussions during courses, responding to participant needs, adjusting the group dynamics, and paying attention to the feedback by course participants.

The Patient School was favorably assessed by participants, staff, and organizers as shown in a previous report by Reinius et al [15]. The perceived value was enhanced by the willingness of peer organizers to share their own experiences, thus creating a sense of belonging and a forum for sharing experiences with like-minded people. In that environment, new knowledge, practical skills, roles, and attitudes were acquired. These experiences felt empowering, and they decreased stigma and reassured participants that one’s identity is not defined by mental health issues.

The thick description of the Patient School based on the comprehensive data reported enables an attempt to present a tentative explanation for these positive outcomes. One way of conceptualizing such a *program theory* is to build on the analysis performed by using the Consolidated Framework for Implementation Research framework [19].

The regional psychiatry organization offered a favorable *outer setting* as demonstrated by its long-term commitment to patient-centered practices and ensuring user influence and involvement. An equally favorable *inner setting* was the outpatient departments providing facilities for the Patient School and allowing their salaried staff with user experience to organize the school, although the lack of dedicated funding and venues was seen as impeding school activities. *Individuals* contributing to the Patient School’s success were the user-involvement coordinators and staff with user experience as course organizers, clinical staff with positive attitudes to user involvement who helped to recruit participants and provide those with information and expert advice, and, finally, service users actively participating and sharing their personal experience. The *implementation process* was guided by the school content, providing practical information on services and support available as well as skills training. The willingness of the course leaders to share their experiences as service users was instrumental to creating a safe environment for participants, enabling them to openly discuss and reflect.

As emphasized in the *Introduction* section, although there are a number of evaluation studies reporting the benefits of recovery colleges and educational activities, implementation processes and experiences are rarely described. However, we find some support for our tentative explanatory model. The *enabling environment* of a recovery college has been said to be a key driver of positive experiences among users and families. Challenges are delays in course standardization and enrollment and attendance procedures. Such barriers can be overcome with a supporting outer setting as well as an inner setting with dedicated staff with user experience and supportive clinical staff [13].

On a more overarching level, the importance of certain characteristics of outer and inner settings has been reported. When assessing several recovery programs, Whitley et al [22] found 4 cross-site themes with an impact on success or failure. They were leadership, organizational culture, training, staff, and supervision. Moreover, they have implications for the implementation process. Other authors highlight the importance of values. Program aims and policies but also practices such as recruitment, staffing, and documentation should be *recovery compatible* [23].

A more practical approach, as used by Smith-Merry et al [24] in Scottish recovery activities, gives useful hints on implementation processes as well. They recommend the application of 4 recovery technologies: recovery narratives (as practiced in the Patient School), the *Scottish Recovery Indicator*, which measures the extent to which services are implementing a recovery-oriented practice model, a structured tool for service users to manage their own recovery, and peer support. While we did not explore the direct influence of the Patient School on the clinical practice, findings indicate that those elements might be found in the Patient School implementation program. The Patient School provided, for example, participants with tools and practices to cope with their challenges and those were assessed in discussions during the sessions. Exchange of lived experience and peer support was a central part of the program.

Finally, not surprisingly, issues on planning and resources are also raised in the literature. Burhouse et al [25] emphasized that when organizing a recovery activity as a continuous improvement, project time for planning is warranted, and *sustainability planning needs resources from the start*. The authors also emphasize the importance of finding a robust measure of the long-term cost-benefit to ensure support from decision makers.

### Strengths and Limitations

This study has strengths as well as weaknesses. It describes a case from 1 psychiatry organization in Sweden and is based on a limited group of interviewees. Attempts to transfer the findings to other contexts must be done with caution [26]. However, this study is focused on the context and processes of implementation, which is an angle seldom chosen in studies evaluating recovery colleges and other educational interventions in psychiatric care. Despite being, in essence, a case study, it adopts a framework, widely used in implementation research, enabling us to present a tentative explanatory model for a recovery college, experienced as being valuable by participants. It shows what features in the context might contribute to the positive impact, as well as the importance of individuals such as organizers with user experience, preconditions in terms of resources, and specifics of the implementation process, the most important being an *open door policy* and *giving everybody space*. Other sites and organizations would be well-advised to pay attention to these features when organizing recovery colleges aiming at strengthening psychiatry service users’ self-management skills and reducing their sense of stigma. Future studies performed in other contexts and comparing different sites would develop and deepen the understanding of the successful implementation of recovery colleges.

### Conclusions

Conditions that will support recovery colleges to reach their goals of empowering psychiatry service users include, first, allocating dedicated resources and engaging, as organizers, individuals with user experience who are willing to share their personal experience. An additional benefit is provided by these organizers working in-house as salaried employees. It is equally important to have an open-door policy, create an open space for participants to share, and offer practical advice and written material that are felt to be useful. Future studies comparing various sites would enhance and broaden our comprehension of the effective implementation of recovery colleges across different contexts.

## Appendix

Multimedia Appendix 1 Timeline of respondent recruitment.

## Abbreviations

COREQ: Consolidated Criteria for Reporting Qualitative Research

## Data Availability

The data sets generated during and analyzed during this study are not publicly available due (due to the nature of this research, interviewees of this study did not agree for their data to be shared publicly) but are available from the corresponding author on reasonable request.
