# Supplementary material for: Implementation of a Recovery College Embedded in a Swedish Psychiatry Organization: Qualitative Case Study
Source: J Particip Med. 2024 Sep 12;16:e55882. doi: 10.2196/55882 (PMC11427861; doi:10.2196/55882)
Supplement: Multimedia Appendix 1 [file jopm_v16i1e55882_app1.docx]

Appendix b. Timeline of respondent recruitment

| **Group of respondents invited** | **N respondents invited** | **N respondents interviewed** | **Method for invitation** | **Time period** |
| --- | --- | --- | --- | --- |
| Participants who had participated in previous PSs (2018-2020) | 39 | 5 | Email | March 2021 |
| Health care professionals who had participated in previous PSs (2018-2020) | 6 | 3 | Email | April 2021 |
| Participants at ongoing PSs | 6 | 3 | [Removed for Peer-review] visited the recovery college | May 2021 |
| Health care professionals who participated in ongoing PSs | 1 | 1 | [Removed for Peer-review] visited the recovery college | May 2021 |
| Leaders of previous and ongoing PSs | 6 | 4 | Email | May 2021 |
